# Near-complete genome sequence of a rhabdovirus identified from the rice pest *Stenchaetothrips biformis*

**DOI:** 10.1128/mra.00001-26

**Published:** 2026-05-18

**Authors:** Lan-Lan Ma, Jun-Min Li, Hai-Jiang Huang

**Affiliations:** 1 State Key Laboratory for Quality and Safety of Agro-Products, Key Laboratory of Biotechnology in Plant Protection of MARA, Zhejiang Key Laboratory of Green Plant Protection, Institute of Plant Virology, Ningbo University 47862, Ningbo, China; Katholieke Universiteit Leuven, Leuven, Belgium

**Keywords:** rhabdoviruses, thrips

## Abstract

The genome sequence of *Stenchaetothrips biformis* rhabdo-like virus (SbRLV) was discovered from the transcriptome sequencing of the rice pest thrips. The 12,436-nt RNA genome potentially encodes four proteins. Phylogenetic analysis groups SbRLV with unclassified thrips-associated rhabdo-like viruses related to *Primrhavirus* and *Alphahymrhavirus*.

## ANNOUNCEMENT

Rhabdoviruses (family *Rhabdoviridae*) are negative-sense single-stranded RNA viruses that infect a wide range of plants and animals (1). Some rhabdovirus species have been reported to deliver genes or double-stranded RNA (dsRNA) into insects, highlighting their potential as genetic tools (2–4). A better understanding of rhabdovirus genomes will facilitate their future utilization. In this study, we report a rhabdo-like virus genome sequence identified from the transcriptome of *Stenchaetothrips biformis*. Specimens of *S. biformis* were originally collected from a rice field in Ningbo, China, and maintained on rice seedlings in the laboratory. Whole insects were grinded in TRIzol reagent (Takara, Dalian, China) to extract total RNA. cDNA library construction was performed by a custom service provided by Novogene (Tianjin, China), as we previously described (5). Paired-end (150 bp) sequencing was conducted on the Illumina NovaSeq 6000 platform. Raw reads were quality checked using FastQC (version 0.11.8), resulting in a total of 22,351,535 clean reads. The clean reads were then *de novo* assembled using Trinity (v2.6.6) (6) with default parameters (--trimmomatic; --min_contig_length 200), yielding 78,361 contigs. These contigs were compared against the NCBI viral RefSeq database (https://www.ncbi.nlm.nih.gov/labs/virus/vssi/; accessed in May 2024) using DIAMOND (v2.0.11.149) BLASTx with an E-value cutoff of 1 × 10^−20^. A near-complete viral genome sequence was identified from this analysis, and tentatively named *Stenchaetothrips biformis* rhabdo-like virus (SbRLV) based on the phylogenetic analysis conducted.

The SbRLV-associated contig was 12,436 nt in length (G + C%: 41.1%), with an average sequencing coverage of 509. Four ORFs were predicted by ORFfinder (Table 1), consistent with Soybean thrips rhabdo-like virus 3 (MW023861.1). Each ORF encodes a protein >250 amino acids, with estimated molecular weights of 28.0–241.1 kDa (DNASTAR v7.1.0). BLASTp analysis yielded significant hits only for ORF1 and ORF4. ORF1 was annotated as a nucleoprotein, which showed highest amino acid similarity with Soybean thrips rhabdo-like virus 3 (71% coverage, 31.2% amino acid identity, as determined by NCBI BLASTp with default parameters). ORF4, encoding an RNA-dependent RNA polymerase (RdRp), is the most conserved. It was best hit by RdRp of Soybean thrips rhabdo-like virus 3 (100% coverage, 50.2% amino acid identity). Noteworthily, while the genome of rhabdoviruses encodes up to a dozen genes, with five conserved across the family (7), the presence of only four ORFs in SbRLV suggests a non-canonical genome organization.

**TABLE 1:** The predicted open reading frames (ORFs) in SbRLV^*a*^

ORF	Annotation	Position	Best match species (Accession)	E-value
ORF1	Nucleoprotein	105–1,835	Soybean thrips rhabdo-like virus 3 (QPZ88389.1)	1e-53
ORF2	Hypothetical protein 1	2,260–4,767	–	–
ORF3	Hypothetical protein 2	4,836–5,588	–	–
ORF4	RNA-dependent RNA polymerase	5,798–1,2199	Soybean thrips rhabdo-like virus 3 (QPZ88392.1)	0.0

^*a*^ "–" indicates that no matching results were found in the BLAST search.

**Fig 1:**
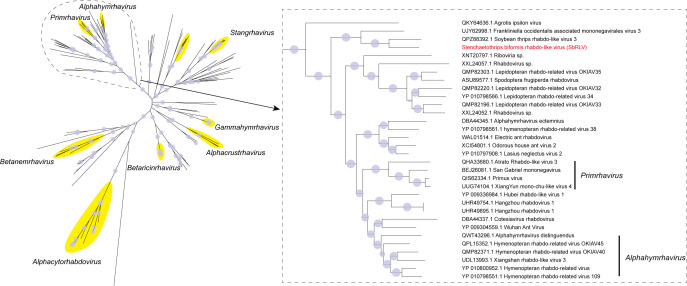
Phylogeny of SbRLV with other related viruses. The maximum likelihood tree was constructed based on conserved RdRp domain sequences of SbRLV and 148 other rhabdoviruses. Sequences were aligned using MAFFT v7.407 (10). The substitution model was evaluated with ModelTest-NG (11), and a maximum likelihood phylogenetic tree was constructed in RAxML-NG (version 0.9.0) (12), with 1,000 bootstrap replicates. Nodes with bootstrap values >50% are marked with solid blue circles, and the larger circles indicate higher bootstrap values. The left panel displays the taxonomic structure of the family Rhabdoviridae, while the right panel provides a detailed view of the clades of interest within the dotted frames. Viruses with an officially classified genus in the NCBI Taxonomy database are highlighted with a yellow background. SbRLV, identified in this study, is marked in red font.

To examine the evolutionary relationships of SbRLV, we performed a maximum-likelihood phylogenetic analysis using an additional 148 RdRp protein sequences from members of rhabdoviruses. The results showed that SbRLV was clustered together with *Frankliniella occidentalis*-associated mononegavirales virus 3 (MW246699.1) and Soybean thrips rhabdo-like virus 3 (8, 9). These thrips-associated rhabdo-like viruses belonged to an unclassified group, which showed a close relationship with genus *Primrhavirus* and *Alphahymrhavirus* (Fig. 1).

This is the first report of a rhabdovirus genome detected in *S. biformis*, thereby expanding the knowledge on rhabdovirus diversity. However, it remains unknown whether SbRLV is an insect-specific virus that restricts to insects or can be transmitted to plants.

## Data Availability

The genome sequence of the SbRLV genomic RNA has been deposited in GenBank under the accession number PX789318. The transcriptome raw sequence data are available in the NCBI Sequence Read Archive under accession SRX31958692.

## References

[1] Walker PJ, Freitas-Astúa J, Bejerman N, Blasdell KR, Breyta R, Dietzgen RG, Fooks AR, Kondo H, Kurath G, Kuzmin IV, Ramos-González PL, Shi M, Stone DM, Tesh RB, Tordo N, Vasilakis N, Whitfield AE, ICTV Report Consortium. 2022. ICTV virus taxonomy profile: rhabdoviridae 2022. J Gen Virol 103:001689. doi:10.1099/jgv.0.001689 35723908 PMC12662027

[2] Gao Q, Xu W-Y, Yan T, Fang X-D, Cao Q, Zhang Z-J, Ding Z-H, Wang Y, Wang X-B. 2019. Rescue of a plant cytorhabdovirus as versatile expression platforms for planthopper and cereal genomic studies. New Phytol 223:2120–2133. doi:10.1111/nph.15889 31059138

[3] Vasilakis N, Tesh RB. 2015. Insect-specific viruses and their potential impact on arbovirus transmission. Curr Opin Virol 15:69–74. doi:10.1016/j.coviro.2015.08.007 26322695 PMC4688193

[4] Kubacki J, Hardmeier I, Qi W, Flacio E, Tonolla M, Fraefel C. 2021. Complete genome sequence of a rhabdovirus strain from Culex Mosquitos collected in Southern Switzerland . Microbiol Resour Announc 10:e01234–20. doi:10.1128/MRA.01234-20 33414339 PMC8407715

[5] Hu TB, Wang XJ, Ye ZX, Lu JB, Li JM, Zhang CX, Huang HJ. 2025. Analysis of salivary proteins in gall-inducing psylla and their potential influence on host plants. BMC Genomics 26:786. doi:10.1186/s12864-025-11958-3 40877775 PMC12395826

[6] Grabherr MG, Haas BJ, Yassour M, Levin JZ, Thompson DA, Amit I, Adiconis X, Fan L, Raychowdhury R, Zeng Q, Chen Z, Mauceli E, Hacohen N, Gnirke A, Rhind N, di Palma F, Birren BW, Nusbaum C, Lindblad-Toh K, Friedman N, Regev A. 2011. Full-length transcriptome assembly from RNA-Seq data without a reference genome. Nat Biotechnol 29:644–652. doi:10.1038/nbt.1883 21572440 PMC3571712

[7] Belot L, Albertini A, Gaudin Y. 2019. Chapter Five - Structural and cellular biology of rhabdovirus entry, p 147–183. *In* Kielian M, Mettenleiter TC, Roossinck MJ (ed), In advances in virus research. Academic Press. 10.1016/bs.aivir.2019.05.003 31439148

[8] Thekke-Veetil T, Lagos-Kutz D, McCoppin NK, Hartman GL, Ju H-K, Lim H-S, Domier LL. 2020. Soybean thrips (Thysanoptera: Thripidae) harbor highly diverse populations of arthropod, fungal and plant viruses. Viruses 12:1376. doi:10.3390/v12121376 33271916 PMC7761488

[9] Chiapello M, Bosco L, Ciuffo M, Ottati S, Salem N, Rosa C, Tavella L, Turina M. 2021. Complexity and local specificity of the virome associated with tospovirus-transmitting thrips species. J Virol 95:e0059721. doi:10.1128/JVI.00597-21 34232724 PMC8513489

[10] Katoh K, Standley DM. 2013. MAFFT multiple sequence alignment software version 7: improvements in performance and usability. Mol Biol Evol 30:772–780. doi:10.1093/molbev/mst010 23329690 PMC3603318

[11] Darriba D, Posada D, Kozlov AM, Stamatakis A, Morel B, Flouri T. 2020. ModelTest-NG: a new and scalable tool for the selection of DNA and protein evolutionary models. Mol Biol Evol 37:291–294. doi:10.1093/molbev/msz189 31432070 PMC6984357

[12] Kozlov AM, Darriba D, Flouri T, Morel B, Stamatakis A. 2019. RAxML-NG: a fast, scalable and user-friendly tool for maximum likelihood phylogenetic inference. Bioinformatics 35:4453–4455. doi:10.1093/bioinformatics/btz305 31070718 PMC6821337

